# Deep Learning–Guided Retinal Vascular Morphometric Quantification in Cerebral Autosomal Dominant Arteriopathy with Subcortical Infarcts and Leukoencephalopathy Mouse Models

**DOI:** 10.1016/j.xops.2026.101279

**Published:** 2026-06-10

**Authors:** Hyungwoo Lee, Na-Kyung Ryoo, Said Arevalo-Alquichire, Kunho Bae, William P. Miller, Joseph F. Arboleda-Velasquez, Leo A. Kim

**Affiliations:** 1 Schepens Eye Research Institute of Massachusetts Eye and Ear and Department of Ophthalmology, Harvard Medical School, Boston, Massachusetts; 2 Department of Ophthalmology, Konkuk University Medical Center, Konkuk University School of Medicine, Seoul, Republic of Korea; 3 Department of Ophthalmology, Seoul National University College of Medicine, Seoul National University Hospital, Seoul, Republic of Korea

**Keywords:** CADASIL, Fluorescein angiography, Explainable artificial intelligence, Diameter, Beading

## Abstract

**Purpose:**

To develop and validate an explainable deep learning–guided workflow to localize and quantify focal retinal luminal pathology on fundus fluorescein angiography (FFA) in NOTCH3 variant knock-in mouse models of cerebral autosomal dominant arteriopathy with subcortical infarcts and leukoencephalopathy.

**Design:**

Cross-sectional experimental imaging and computational analysis.

**Subjects:**

Thirty-two mice (wild-type [WT] n = 12; NOTCH3^C455R^ [C455R] n = 12; NOTCH3^R1031C^ [R1031C] n = 8) yielding 1670 analyzable FFA images.

**Methods, Intervention, or Testing:**

Two ImageNet-pretrained VGG16 classifiers (WT vs. each mutant line) were trained with subject-grouped splitting. Grad-CAM++ and occlusion sensitivity maps defined class-discriminative regions of interest (ROIs). A centerline-based morphometry pipeline sampled luminal diameter along ordered vessel centerlines to compute mean and maximum diameter, diameter coefficient of variation, and tortuosity. A fast Fourier transform–derived vessel beading index (VBI) quantified periodic diameter oscillations using normalized, band-limited spectral power. Metrics were computed for large-vessel and small-vessel masks in both whole-field and ROI-restricted domains. Additional robustness analyses assessed hold-out testing, out-of-sample saliency, ROI-threshold sensitivity, alternative VBI spatial-period bands, and repeated balanced retraining.

**Main Outcome Measures:**

Primary biological outcomes were large-vessel mean diameter, maximum diameter, and VBI in whole-field and ROI-restricted analyses; classifier discrimination (area under the curve) was reported as supportive performance of the localization framework.

**Results:**

Whole-field morphometry detected generalized large-vessel dilation in both mutants versus WT (mean diameter: WT 27.82 μm; C455R 30.94 μm; R1031C 32.03 μm; *P* ≤ 0.001) with reduced tortuosity (*P* < 0.001), whereas whole-field maximum diameter and VBI increased only directionally. ROI-restricted analysis amplified focal pathology: within Grad-CAM++ ROIs, large-vessel maximum diameter increased (WT 37.62 μm; C455R 48.46 μm; R1031C 45.66 μm; *P* < 0.001) and VBI increased (WT 1.75; C455R 3.04; R1031C 2.93; *P* ≤ 0.001). Occlusion ROIs showed concordant VBI increases (WT 2.11; C455R 3.84; R1031C 4.90; *P* < 0.001). Small-vessel ROI differences were minimal. The principal large-vessel ROI-restricted phenotype remained directionally stable across robustness analyses, and hold-out testing confirmed high classifier discrimination in both genotype comparisons.

**Conclusions:**

Explainable deep learning–guided localization on FFA identifies disease-informative vessel segments and enables sensitive quantification of focal luminal dilation and periodic beading that are diluted by whole-field averages. This framework may support development of retinal biomarkers and longitudinal monitoring in cerebral autosomal dominant arteriopathy with subcortical infarcts and leukoencephalopathy.

**Financial Disclosures:**

Proprietary or commercial disclosure may be found in the Footnotes and Disclosures at the end of this article.

Cerebral autosomal dominant arteriopathy with subcortical infarcts and leukoencephalopathy (CADASIL) is an inherited small-vessel disease caused by pathogenic *NOTCH3* mutations. In CADASIL, these mutations are associated with the deposition of granular osmiophilic material (GOM) and degeneration of vascular smooth muscle cells (VSMCs) and pericytes in small arteries, leading to progressive arteriopathy and ischemic injury in the brain and other organs.^1^ Histologic studies have documented vessel wall thickening and GOM deposits in retinal vessels, paralleling the pathological changes seen in cerebral arteries.^2^ Clinically, retinal imaging findings in CADASIL have been heterogeneous, ranging from arteriolar narrowing and arteriovenous nicking to subtle or undetectable changes.^3^, ^4^, ^5^, ^6^, ^7^ However, it remains unclear which specific morphological metrics best capture the dynamic luminal pathology inherent to the disease.

A key challenge is that many existing retinal vascular metrics are derived from fundus photography and rely on descriptive or optic disc-centered sampling schemes (e.g., concentric zones around the optic disc).^3^, ^4^, ^5^, ^6^ These measurements are inherently limited by technical artifacts because color fundus photography defines apparent vessel borders from reflectance-based intensity gradients, and the central light reflex can bias diameter measurements.^8^ Furthermore, disc-centric indices summarize diameter only near the optic disc and typically sample a limited number of locations.^9^ As a result, they may be relatively insensitive to focal or segment-specific diameter abnormalities, such as the segmental diameter irregularity with alternating constrictions and dilations.

Fundus fluorescein angiography (FFA) offers a complementary perspective by delineating the intraluminal dye column, providing high contrast for both large and small vessels and a more direct readout of luminal diameter than reflectance images.^10^ However, capturing irregular luminal morphology requires analysis beyond average diameter. Sampling diameter along the vessel centerline yields an along-track diameter trace that captures subtle fluctuations along the vessel length. Frequency-domain analysis then converts these spatial variations into spectral power, enabling objective quantification of periodic constrictions (beading pattern) that may be underestimated by simple summary statistics such as the mean or standard deviation.^11^^,^^12^

Even with precise centerline measurements, whole-field analysis across the entire retinal network can mask focal pathological signals because global averaging dilutes disease-informative morphological signatures. This suggests that identifying where to look is as critical as what to measure. Recent advances in deep learning offer a potential solution. Convolutional neural networks can learn subtle vascular image features that are difficult to hand-engineer, while explainable artificial intelligence (XAI) and perturbation-based methods such as Grad-CAM++ and occlusion sensitivity can highlight image regions that most strongly influence model predictions.^13^^,^^14^ These types of deep learning approaches have been applied across diverse biomedical imaging tasks, including angiographic and vascular imaging modalities.^15^, ^16^, ^17^ We hypothesized that by focusing quantitative morphometry on these artificial intelligence–highlighted regions, we could unmask focal pathological features that are otherwise diluted in whole-field analyses.

Here, we aim to identify interpretable luminal markers of CADASIL in retinal vessels and to illustrate how XAI can help focus quantitative analysis on the most informative regions. We combined FFA, along-track luminal morphometry, and deep learning–guided region of interest (ROI) selection to characterize CADASIL-related retinal vascular remodeling in vivo in 2 Notch3 knock-in mouse lines *(NOTCH3*^C455R^ and *NOTCH3*^R1031C^), which represent more severe and milder hypomorphic Notch3 activity states, respectively.^18^^,^^19^ We trained a VGG16-based convolutional neural network classifier to distinguish FFA images from wild-type (WT) and mutant mice, applied Grad-CAM++ with complementary occlusion sensitivity to derive class-discriminative ROIs, and then performed quantitative morphometry within the whole vascular field and within vessels intersecting the XAI-defined ROIs. Endpoints included mean and maximum luminal diameter, diameter coefficient of variation (CV), a Fourier-based vessel beading index (VBI) derived from the centerline radius trace, and tortuosity. We first compared WT and mutants in whole-field analysis and then tested whether XAI-restricted ROIs reveal amplified genotype differences, thereby assessing whether the model attends to biologically relevant vascular pathology in CADASIL.

## Materials and Methods

### Animals

All animal procedures were approved by the Institutional Animal Care and Use Committee of Massachusetts Eye and Ear (protocol no. 2021N000163) and were conducted in accordance with the National Institutes of Health Guide for the Care and Use of Laboratory Animals and the Association for Research in Vision and Ophthalmology Statement for the Use of Animals in Ophthalmic and Vision Research. We studied 2 CADASIL human *NOTCH3* variant knock-in lines (C455R and R1031C) on an endogenous Notch3 knockout background under the control of SM22α-Cre. Mutant cohorts were generated by crossing human *NOTCH3*^C455R^ knock-in line (B6;129*Gt(ROSA)26Sor*^*tm2(NOTCH3C455R)Sat*^/Mmjax) or the human *NOTCH3*^R1031C^ knock-in line (B6;129S*Gt(ROSA)26Sor*^*tm1(NOTCH3*R1031C)Sat^/Mmjax) with Notch3 knockout SM22-Cre mice (hereafter referred to as C455R and R1031C mice).^18^^,^^19^ Because the mutant lines were maintained on a C57BL/6 background, age-matched C57BL/6J mice (The Jackson Laboratory; stock no. 000664) were used as WT controls. WT and C455R cohorts were imaged at 6 months, whereas R1031C mice were imaged at 8 months because pathology is less aggressive and due to experimental scheduling and animal availability. Sex distribution differed across cohorts (WT: 12M/0F; C455R: 9M/3F; R1031C: 2M/6F, Supplemental Table 1 available at www.ophthalmologyscience.org). Because the WT cohort was male-only and the R1031C cohort was older and more female-predominant, genotype contrasts—particularly WT versus R1031C—should be interpreted as biologically informative but potentially confounded by age/sex differences. Because this was an animal-only experimental study and did not involve human participants or identifiable human data, institutional review board approval, adherence to the Declaration of Helsinki, and informed consent were not applicable.

### In Vivo Imaging

Fundus fluorescein angiography images were acquired from both eyes using a Micron IV system (Phoenix Research Labs). Mice were anesthetized with ketamine/xylazine (12.5/2.5 mg/mL; 0.16 mL/20 g, intraperitoneal), pupils were dilated with 1% tropicamide, and the cornea was lubricated during imaging. Angiography was performed after intraperitoneal injection of 0.05 mL of 25% fluorescein sodium. For each mouse, both eyes were imaged sequentially across angiography phases (early to late) using the same acquisition settings. High-quality images were selected, excluding motion, saturation, or blink artifacts. Images were acquired at 800 × 800 pixels, and pixel distances were approximated to micrometers using a nominal scale factor based on the manufacturer-reported mouse field of view (1.8 mm across 50°), corresponding to 2.25 μm/pixel (1.8 mm/800 pixels).

### Vessel Mask Generation–Large and Small Vessels

**Figure 1:**
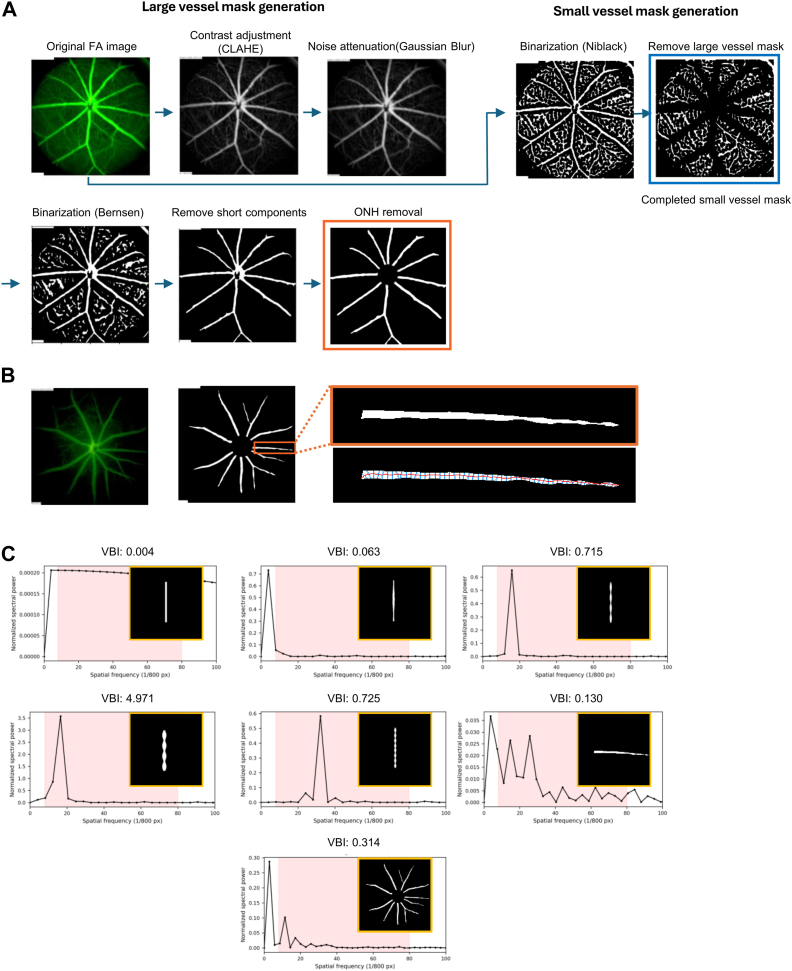
Analysis pipeline for centerline-based morphometry and FFT-based vessel beading quantification. (**A**) Preprocessed FFA images were used to generate binary masks for large vessels (Bernsen thresholding) and small vessels (Niblack thresholding). (**B**) Vessel masks were thinned to one-pixel-wide centerlines (red line) and split at branch points into nonbranching segments. Diameters sampled along each segment centerline (blue lines) were used to compute mean diameter, maximum diameter, diameter coefficient of variation (CV), and tortuosity. (**C**) Fourier-based vessel beading index (VBI). Along-track diameter traces were transformed into the frequency domain (power spectral density, PSD). VBI was defined as the integrated PSD power within a target spatial-period band (10–100 pixels, red shading) normalized by N^2^, where N is the number of valid diameter samples per segment, to remove length bias. Segment-level VBI values were combined using length-weighted averaging. Examples illustrate that focal beading increases power within this band. A rectangular diameter profile (top left) shows no band-limited peak and does not increase VBI. A long-period, slowly varying bulge (top middle) produces power predominantly outside the predefined 10 to 100 pixel band and therefore does not increase VBI. In contrast, a beading-like oscillatory pattern (top right) yields a spectral peak within the red band, increasing VBI. Increasing beading amplitude (second row, left) increases band-limited power and therefore increases VBI. A change in oscillation period alone without a corresponding increase in band-limited power (second row, middle) does not necessarily increase VBI. The second-row right panel shows an example PSD derived from an individual vessel segment, and the bottom row shows an example of VBI computation when the full large-vessel mask is used as input. FFT = fast Fourier transform.

For FFA images, we generated binary masks for large vessels (major arterioles/venules; trunks/first-order branches) and small vessels (higher-order branches) (Fig 1A). Images were converted to grayscale by extracting the green channel in ImageJ. Local contrast enhancement was then performed in Python using contrast-limited adaptive histogram equalization implemented with skimage.exposure.equalize_adapthist (clip_limit = 0.01). The contextual region (kernel_size) was not manually overridden; thus, for 800 × 800 images, the default contextual region corresponded to approximately 100 × 100 pixels. The preprocessed images were denoised with a Gaussian blur (σ = 1 pixel). Large vessels were segmented using Bernsen local thresholding (radius 15 pixels),^20^ skeletonized, and fragments shorter than 20 pixels (∼45 μm) were removed to retain major trunks and first-order branches. For large-vessel morphometry, a single binarized image was selected for each eye based on prespecified quality criteria prioritizing vessel continuity, adequate focus, minimal motion/blink artifact, minimal saturation or obscuration, and minimal contamination from small-vessel signal. A circular region corresponding to the optic nerve head was excluded. Small vessels were segmented from the original FFA images using Niblack local thresholding (radius 15 pixels).^21^ The large-vessel mask (prior to optic nerve head exclusion) was subtracted to yield the small-vessel network composed of precapillary arterioles and capillaries. For small-vessel morphometry, one binarized image per eye was similarly selected using the same quality criteria while prioritizing clear delineation of the higher-order vascular network and minimal dominance of large-vessel signal. Image selection was performed while masked to genotype. Green-channel extraction and local thresholding were performed in ImageJ (v1.53; National Institutes of Health), whereas contrast-limited adaptive histogram equalization preprocessing and subsequent binary-mask cleanup steps were performed in Python.

### Centerline-Based Morphometry and Length-Weighted Aggregation

Large-vessel and small-vessel binary masks were thinned to one-pixel-wide centerlines and split at branchpoints into nonbranching segments. Centerline coordinates within each segment were ordered along the vessel path using a graph traversal implemented in Python (Fig 1B). Vessel radii were obtained from the Euclidean distance transform of the binary vessel mask and sampled along the ordered centerline, and diameters were computed as 2 × radius (Fig 1B).^22^ For each image, we computed mean diameter, maximum diameter, diameter CV (pooled standard deviation of all sampled diameters divided by the length-weighted mean diameter), and tortuosity (path length/chord length). Segment-level quantities were aggregated to image-level values using length-weighted averaging (weights proportional to segment length) to reduce sensitivity to short fragmented segments. These steps were implemented in a custom Python pipeline using scikit-image (v0.19.3), NumPy (v1.21.5), and SciPy (v1.7.3).

### Fourier-Based Vessel Beading Index

To quantify periodic constrictions and dilations along vessels (beading), we analyzed the along-track diameter trace of each segment in the frequency domain using the fast Fourier transform (FFT) similar to previous studies.^11^^,^^12^ Unlike dispersion-based summaries such as the diameter CV, which capture overall variability without regard to spatial ordering, the FFT decomposes the diameter trace into spatial-frequency components and thereby emphasizes structured, periodic oscillations along the vessel. For each segment, we treated the ordered diameter samples as a one-dimensional spatial signal and computed its Fourier transform. The squared magnitude of the Fourier coefficients yields a power spectrum (power spectral density), which summarizes how strongly diameter fluctuations occur. Based on empirical inspection of bead spacing in our 800 × 800 pixel data set and consensus among clinical investigators (H.L., N.R., and L.A.K.), we focused on a target spatial-period band of 10 to 100 pixels to capture disease-relevant beading while reducing contributions from very low-frequency global trends and high-frequency pixel-level noise.

Because the summed FFT power scales with the number of samples (segment length), we used a length-bias–corrected VBI by normalizing the band-limited power by the square of the segment length (N^2^, where N is the number of valid diameter samples), enabling comparison across segments of different lengths. Segment-level VBI values were then aggregated into an eye-level metric using length-weighted averaging across all valid segments. Representative examples are shown in Figure 1C.

### Deep Learning Classifier

We fine-tuned 2 ImageNet-pretrained VGG16 models^23^^,^^24^ (WT vs. C455R and WT vs. R1031C) to classify individual FFA images as WT versus mutant using PyTorch (v2.1) on an NVIDIA GeForce RTX 3080 GPU. A fixed random seed (42) was set prior to data splitting and model initialization. For each comparison, the classification data set comprised all eligible FFA images acquired from both eyes across early-to-late angiographic phases after exclusion of poor-quality images as defined above (Supplemental Table 1). To reduce the influence of acquisition overlays, 2 rectangular regions in the upper-left and lower-left corners (170 × 20 pixels) were replaced with random noise. This preprocessing was applied identically to training, validation, and test images. Images were converted to 3 channels and resized to 256 × 256 pixels. Training-time augmentation included random resized cropping (scale 0.9–1.0), brightness/contrast jitter (0.2), random affine transformation (rotation ±15°, translation ±10%, scaling 0.9–1.1), and horizontal flipping.

VGG16 was selected as a pragmatic baseline for this moderate-sized data set because it provides a widely used convolutional architecture with straightforward computation of Grad-CAM++ saliency maps.^23^

To prevent data leakage, splitting was performed at the mouse level such that all images from a given mouse were confined to a single partition. Fifteen percent of mice were held out as an independent test set. The remaining mice were used in 5-fold subject-grouped cross-validation. Within each fold, models were trained using focal loss (γ = 2) with class-balanced weights derived from the training fold and optimized with Adam (learning rate 1 × 10^–4^; weight decay 1 × 10^–5^), using batch size 32 and early stopping (patience 5; maximum 100 epochs). For receiver operating characteristic/area under the curve (AUC) evaluation, predictions from the 5-fold–specific models were combined to form ensemble classifiers using soft voting, hard voting, and weighted soft voting. AUCs were computed at the mouse level by averaging predicted probabilities across all images from the same mouse before computing AUC across mice.

### Saliency Maps, Ensemble Aggregation, and Region of Interest Definition

**Figure 2:**
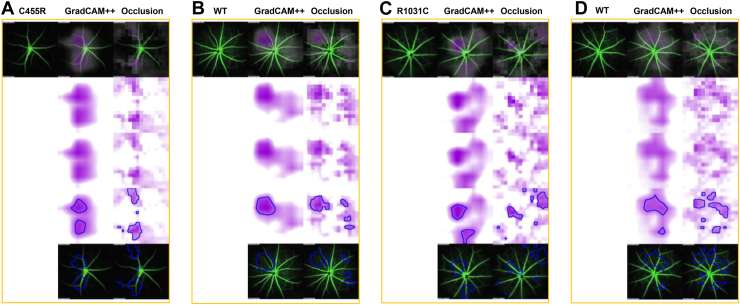
Workflow for deriving XAI-based vessel regions of interest (ROIs). Representative examples illustrating generation of analysis masks from saliency maps. Top row: Original FFA with overlaid Grad-CAM++ and occlusion sensitivity maps. Middle row: Raw saliency maps (percentile-normalized to 0–1). Third row: Blue contours delineate the top 10% high-attention regions. Bottom row: Final XAI-restricted ROIs (blue contours) overlaid on original images. Intersections of saliency maps and vessel masks define XAI-restricted ROIs used for focal morphometry. Examples show (**A**) C455R, (**B**) WT (C455R-classifier), (**C**) R1031C, and (**D**) WT (R1031C-classifier). FFA = fundus fluorescein angiography; WT = wild-type; XAI = explainable artificial intelligence.

To interpret the classifier’s decision basis, we applied 2 complementary saliency methods, a gradient-based method (Grad-CAM++) and a perturbation-based method (occlusion sensitivity).^13^^,^^14^ For Grad-CAM++, class-discriminative heatmaps were computed from the last convolutional block of each of the 5-fold–specific VGG16 models, resized to the original FFA resolution, minimum to maximum normalized to the 0 to 1 range within each image, and averaged pixelwise across folds to obtain an ensemble Grad-CAM++ map per image (Fig 2). Occlusion sensitivity was computed by sliding a 15 × 15–pixel window with 15-pixel stride and recording the change in the mutant-class probability, and fold-level occlusion maps were similarly normalized and averaged to obtain an ensemble occlusion map (Fig 2).

For each genotype contrast and each XAI method, ensemble saliency maps were thresholded at the 90th percentile (top 10%) to define binary XAI ROIs. The 90th-percentile threshold was retained as a pragmatic primary setting that focused analysis on the highest saliency regions while retaining sufficient vessel coverage for stable ROI-restricted morphometry, and the final out-of-sample threshold-sensitivity analyses were used to confirm that the principal large-vessel phenotype was not dependent on that single cutoff. Each ROI was intersected with the corresponding large-vessel or small-vessel mask, yielding whole-field vessels and ROI-restricted vessels for morphometry (Fig 2). Because saliency maps can be sensitive to modeling choices and do not necessarily imply causal image evidence,^25^^,^^26^ we treated saliency as hypothesis-generating and prioritized endpoints that were consistent across both Grad-CAM++ and occlusion ROIs, in addition to whole-field analysis.

To assess whether ROI-restricted morphometry depended on in-sample saliency estimation, we additionally recalculated saliency maps under an out-of-sample policy. Test-set mice were evaluated with all 5 saved fold-specific models, whereas train/CV mice were evaluated only with the saved model from the fold in which that mouse served as validation. Accordingly, each saliency map in this analysis was generated only with models that had not been trained on the corresponding mouse.

### Region of Interest–Restricted Morphometry and Statistical Analysis

Quantitative outcomes are summarized as subject-level means ± standard deviation. In line with the biological emphasis of the study, large-vessel mean diameter, maximum diameter, and VBI were predefined as the principal morphometric endpoints of interest. The remaining measures and the additional robustness analyses were treated as secondary or supportive sensitivity analyses. Between-group comparisons are reported with group means and 2-sided *P* values, and we additionally report absolute mean differences (mutant minus WT) with bootstrap 95% confidence intervals (CIs) as effect estimates on the original scale for the primary whole-image and ROI-restricted analyses and for the key robustness analyses. Because group sizes were small and distributions may deviate from normality, we used the nonparametric Mann–Whitney U test (WT vs. each mutant). Given the study design involving multiple comparisons against the same control group (WT vs. C455R and WT vs. R1031C), we conservatively applied a Bonferroni correction, setting the significance threshold at α = 0.025 (0.05/2). Bootstrap CIs were obtained by subject-level resampling with replacement using 5000 bootstrap replicates. We retained the Mann–Whitney U test as the prespecified distribution-robust hypothesis test, while reporting mean differences with bootstrap CIs as clinically interpretable effect estimates on the original scale.

To assess dependence on the ROI definition, we repeated the ROI-restricted analysis on the final out-of-sample manifest while varying only the saliency threshold (80th, 85th, 90th, 95th, and 97.5th percentiles), with the VBI definition fixed at the primary 10 to 100 pixel band. These analyses are summarized as subject-level means ± standard deviation, mean differences (mutant minus WT) with bootstrap 95% CIs, and exact 2-sided *P* values. Bootstrap CIs were obtained by subject-level resampling with replacement using 5000 bootstrap replicates. We retained the Mann–Whitney *U* test as a distribution-robust hypothesis test, while reporting mean differences with bootstrap CIs as clinically interpretable effect estimates on the original scale. To assess dependence on the VBI definition, we repeated the VBI analysis on the same final out-of-sample manifest while fixing the primary XAI ROI threshold at the 90th percentile and recalculating VBI across alternative spatial-period bands (5–150, 15–100, and 20–80 pixels), alongside the primary 10 to 100 pixel band.

To evaluate the effect of image-count imbalance in the WT-versus-R1031C comparison, we performed repeated balanced retraining within the original mouse-level grouped split and fixed independent hold-out test set. For each repeat and each fold, only the WT training pool was downsampled to match the number of R1031C training images; validation and test sets were left unchanged. Downsampling was subject-aware and random, with repeat-specific and fold-specific fixed seeds. Fold-specific models were retrained under the same preprocessing, augmentation, and optimization framework described above, and repeat-wise ensemble performance was summarized on the fixed independent test set.

Morphometric analysis was performed on selected binarized images at the eye level, with one image per eye for large-vessel analysis and one image per eye for small-vessel analysis. Eye-level values were then averaged to derive subject-level summaries, and the primary statistical comparisons were performed at the subject level.

Statistical analyses were performed in Python using SciPy (v1.7.3).

## Results

### Data Set Characteristics and Deep Learning Classifier Performance

**Figure 3:**
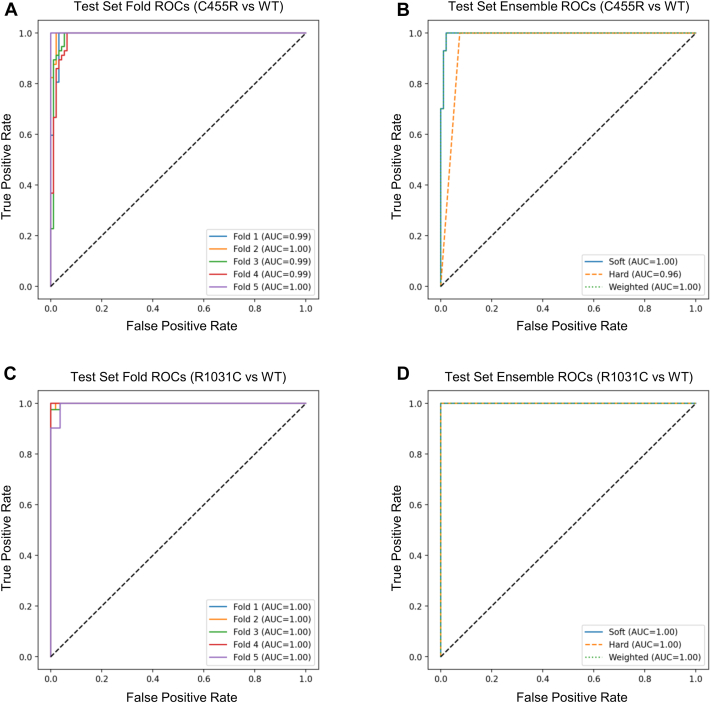
Performance of VGG16 classifiers for CADASIL versus WT. Receiver operating characteristic (ROC) curves for (**A**, **B**) C455R versus WT and (**C**, **D**) R1031C versus WT. Panels **A** and **C** show ROC curves for individual subject-grouped cross-validation folds. Panels **B** and **D** show ensemble ROC curves on the independent test set using soft voting, hard voting, and weighted soft voting. AUCs were computed at the mouse level by averaging predicted probabilities across all images from the same mouse. Overall, classifiers showed good discrimination of mutant genotypes from controls. On the independent hold-out test set, mouse-level soft-voting AUC was 1.00 in both genotype comparisons, whereas image-level soft-voting AUC was 0.984 for WT versus C455R and 1.00 for WT versus R1031C. AUC = area under the curve; CADASIL = cerebral autosomal dominant arteriopathy with subcortical infarcts and leukoencephalopathy; WT = wild-type.

The final data set included 32 mice (WT, n = 12; C455R, n = 12; R1031C, n = 8), yielding 1670 analyzable FFA images (Supplemental Table 1). Under subject-grouped evaluation, VGG16 classifiers showed high discrimination of each mutant line versus WT across individual cross-validation folds (Fig 3A, C). We additionally reconstructed the original mouse-level hold-out split and re-evaluated classifier performance using only the independent test-set mice. In the C455R comparison, image-level soft-voting AUC was 0.984 and mouse-level soft-voting AUC was 1.00. In the R1031C comparison, both image-level and mouse-level soft-voting AUCs were 1.00. Because the independent test partitions were small, these analyses were interpreted as direct out-of-sample confirmation rather than high-precision estimates of generalization performance (Supplemental Table 2, available at www.ophthalmologyscience.org).

### C455R versus Wild-Type

**Figure 4:**
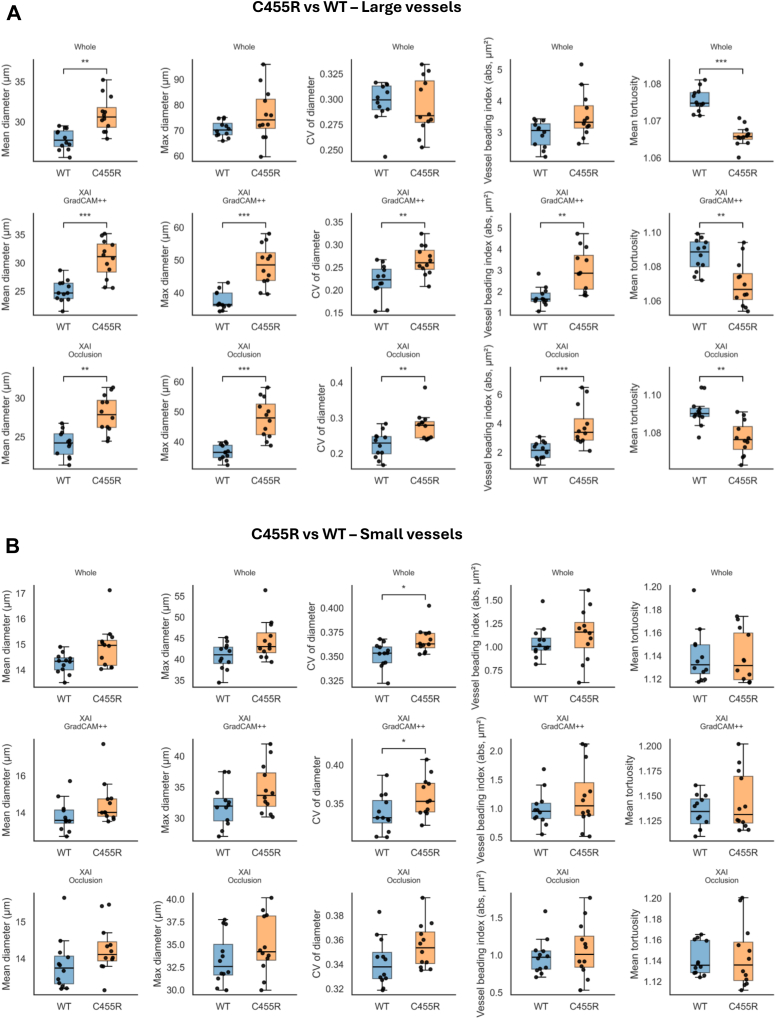
Quantitative vascular metrics in C455R versus WT. Box-and-whisker plots of subject-level morphometry for large and small vessels across 3 domains: Whole image, Grad-CAM++ ROIs, and occlusion ROIs. (**A**) In large vessels, whole-image analysis detects global dilation and reduced tortuosity in C455R, whereas XAI-restricted analyses (Grad-CAM++ and occlusion) reveal increased maximal diameter, diameter CV, and vessel beading index in focal segments. (**B**) Small-vessel effects were modest overall. Whole-image diameter CV was modestly increased in C455R, whereas ROI-restricted small-vessel differences did not meet the Bonferroni-corrected threshold. Significance was determined by Mann–Whitney U tests with Bonferroni correction (∗*P* < 0.025, ∗∗*P* < 0.01, ∗∗∗*P* < 0.001). CV = coefficient of variation; ROI = region of interest; WT = wild-type; XAI = explainable artificial intelligence.

In whole-image analysis, C455R mice showed global dilation relative to WT in large vessels (Fig 4, Supplemental Table 3, available at www.ophthalmologyscience.org), with increased mean diameter (WT 27.82 μm vs. C455R 30.94 μm; *P* = 0.001) and reduced tortuosity (1.08 vs. 1.07; *P* < 0.001) in C455R, consistent with vessel straightening. In contrast, maximum diameter and diameter CV did not differ significantly, and VBI showed a nominal increase but did not meet the Bonferroni-corrected threshold (*P* = 0.040; Supplemental Table 3). In small-vessel masks, mean diameter was marginally higher in C455R but not statistically significant (14.26 μm vs. 14.90 μm; *P* = 0.030), whereas diameter CV was higher in C455R (0.35 vs. 0.37, *P* = 0.017).

**Figure 5:**
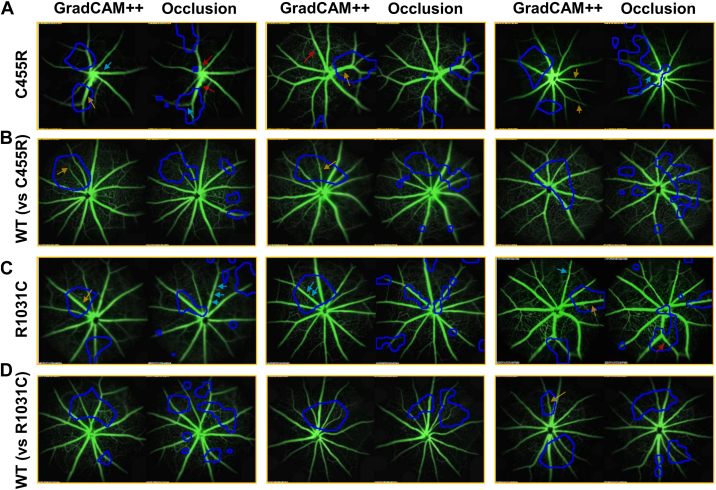
XAI-derived vessel ROIs in CADASIL mutants and controls. Representative overlays of XAI-restricted ROIs (blue contours) on FFA images. (**A**) C455R. In the first panel, the Grad-CAM++ ROI highlights a thickened vessel segment (orange arrow), while periodic beading of a large vessel is also visible outside the ROI (blue arrow). The occlusion ROI overlaps a similar region and again captures a beading pattern (blue arrow), and additional focal diameter enlargement is seen in other areas (red arrows). In the second panel, the Grad-CAM++ map highlights a segment showing diameter variation with both widening and focal narrowing, and diameter changes are also noted outside the ROI (red arrow). The occlusion map points to a similar region. In the third panel, a vessel at approximately the 9:30 o’clock direction shows a characteristic beading pattern within regions highlighted by both Grad-CAM++ and occlusion (blue arrow), while milder beading is also present in thinner vessels elsewhere (orange arrows). (**B**) WT control (tested against C455R). ROIs are sparse and primarily encompass relatively smooth vessels. Increased tortuosity is observed within the XAI-highlighted region (orange arrow). (**C**) R1031C. In the first panel, both XAI methods highlight a dilated vessel segment (orange arrow), and a beading pattern is also apparent in a vessel near the 1 o’clock position (blue arrows). In the second panel, a vessel highlighted by both methods shows repetitive periodic undulations (blue arrows). In the third panel, the vessel indicated by both XAI methods near the 3 o’clock direction demonstrates diameter irregularity (orange arrow), with a focal dilation near the 5:30 o’clock position (red arrow); mild beading is also visible outside the Grad-CAM++ ROI (blue arrow). (**D**) WT control (tested against R1031C). ROIs again predominantly encompass smooth vessels with minimal diameter fluctuation. In the third panel, mild tortuosity is noted within the region highlighted by both XAI methods (orange arrow). CADASIL = cerebral autosomal dominant arteriopathy with subcortical infarcts and leukoencephalopathy; FFA = fundus fluorescein angiography; ROI = region of interest; WT = wild-type; XAI = explainable artificial intelligence.

In XAI-restricted ROIs (Grad-CAM++ and occlusion), saliency maps highlighted focal large-vessel regions with pronounced focal pathology in large vessels (Fig 2). Within Grad-CAM++ large-vessel ROIs, C455R demonstrated focal dilation relative to WT with increased mean diameter (25.04 μm vs. 30.70 μm; *P* < 0.001), similar to the whole-image analysis. Unlike the whole-image analysis, C455R also showed increased maximum diameter within Grad-CAM++ ROIs (37.62 μm vs. 48.46 μm; *P* < 0.001) (Figs 4A and 5A, B, Supplemental Table 4, available at www.ophthalmologyscience.org). Diameter CV (0.22 vs. 0.27; *P* = 0.006) and VBI (1.75 vs. 3.04; *P* = 0.001) were also increased in these ROIs, indicating preferential sampling of segments with periodic beading and irregular diameter (Figs 4A and 5A, B, Supplemental Table 4). Occlusion-defined ROIs showed a concordant pattern for focal dilation and beading metrics (Fig 5A, B, Supplemental Table 5, available at www.ophthalmologyscience.org). Overall, XAI-restricted analysis highlighted focal segments in large vessel masks in which genotype differences were largest.

In contrast, small vessels within XAI-restricted ROIs showed limited effects. Small-vessel metrics showed directionally similar differences relative to WT but did not reach statistical significance (Fig 4B, Supplemental Tables 4 and 5).

Under the out-of-sample saliency policy, the principal large-vessel phenotype remained evident in C455R. In Grad-CAM++ large-vessel ROIs, C455R remained higher than WT in mean diameter, maximum diameter, and VBI, and occlusion-defined ROIs showed the same directional pattern. Small-vessel VBI differences were not robust (Supplemental Table 6, available at www.ophthalmologyscience.org).

On the final out-of-sample manifest, large-vessel threshold sensitivity was strongest in Grad-CAM++ ROIs. Across thresholds 80 to 97.5, large-vessel mean diameter, maximum diameter, and VBI remained consistently higher in C455R than in WT. Occlusion-defined large-vessel ROIs showed the same directional pattern, with VBI strongest at lower-to-intermediate thresholds (Supplemental Table 7, available at www.ophthalmologyscience.org).

With the XAI ROI threshold fixed at the primary 90th-percentile setting on the final out-of-sample manifest, large-vessel VBI remained higher in C455R than in WT across all tested spatial-period bands. Support was strongest in Grad-CAM++ large-vessel ROIs, whereas occlusion-defined large-vessel ROIs showed a concordant directional pattern across bands. Small-vessel VBI differences remained minimal (Supplemental Table 8, available at www.ophthalmologyscience.org).

### R1031C versus Wild-Type

**Figure 6:**
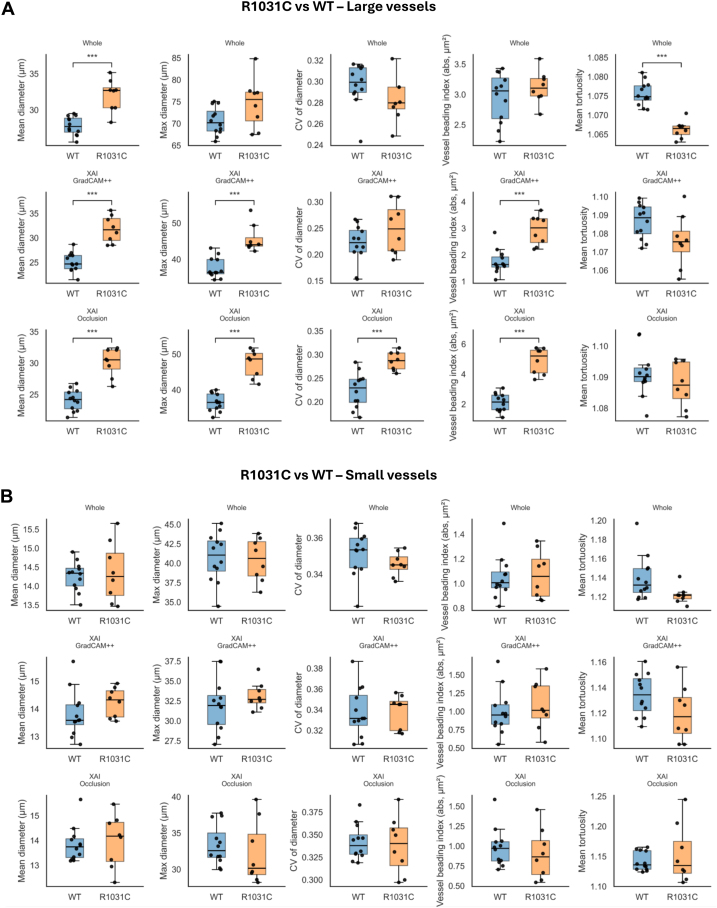
Quantitative vascular metrics in R1031C versus WT. Box-and-whisker plots comparing WT and R1031C mice. (**A**) Large vessels: Whole-image analysis shows dilation and reduced tortuosity, whereas XAI-restricted ROIs amplify the vessel beading index (VBI) signal in focal segments. Diameter CV showed method-dependent significance across XAI definitions, whereas VBI was elevated across both. (**B**) Small vessels: No robust differences were observed. ∗*P* < 0.025, ∗∗*P* < 0.01, ∗∗∗*P* < 0.001; Mann–Whitney U test with Bonferroni correction. CV = coefficient of variation; ROI = region of interest; WT = wild-type; XAI = explainable artificial intelligence.

Whole-image analysis similarly showed global large-vessel dilation in R1031C relative to WT (Fig 6A, Supplemental Table 3), with increased mean diameter (WT 27.82 μm vs. R1031C 32.03 μm; *P* < 0.001) and reduced tortuosity (1.08 vs. 1.07; *P* < 0.001). Whole-image maximum diameter, diameter CV, and VBI were not significantly different. In small vessels, whole-image metrics showed no significant deviations from WT, suggesting limited small-vessel remodeling at this age.

In XAI-restricted ROIs (Grad-CAM++ and occlusion) in large vessels, focal dilation and beading were observed. In Grad-CAM++ large-vessel ROIs, R1031C showed significant focal increases in mean (25.04 μm vs. 31.80 μm; *P* < 0.001) and maximum diameter (37.62 μm vs. 45.66 μm; *P* < 0.001) and VBI (1.75 vs. 2.93; *P* < 0.001) (Figs 5C, D and 6A, Supplemental Table 4). This focal phenotype was corroborated in occlusion-defined ROIs, with significant increases in mean and maximum diameter, diameter CV, and VBI (Supplemental Table 5).

For small vessels, however, R1031C did not show robust separation from WT across both XAI methods in terms of VBI or related metrics (Fig 6B, Supplemental Tables 4 and 5).

Under the out-of-sample saliency policy, the principal large-vessel phenotype also remained evident in the R1031C cohort. In Grad-CAM++ large-vessel ROIs, R1031C remained higher than WT in mean diameter, maximum diameter, and showed a concordant VBI increase. In occlusion-defined large-vessel ROIs, mean diameter, maximum diameter, diameter CV, and VBI all remained elevated in R1031C relative to WT, whereas small-vessel VBI differences were not robust (Supplemental Table 6).

Threshold sensitivity in the R1031C cohort again localized primarily to large-vessel ROIs. In Grad-CAM++ large-vessel ROIs, mean and maximum diameter remained significantly higher in R1031C than in WT across all tested thresholds, and VBI remained directionally consistent across all thresholds. In occlusion-defined large-vessel ROIs, mean diameter, maximum diameter, and VBI all remained significantly higher in R1031C than in WT across the full threshold range (Supplemental Table 9, available at www.ophthalmologyscience.org).

We additionally examined VBI sensitivity to alternative spatial-period bands in the R1031C cohort while fixing the ROI threshold at the primary 90th percentile. In occlusion-defined large-vessel ROIs, VBI remained consistently higher in R1031C than in WT across all tested bands, including 10 to 100, 15 to 100, 20 to 80, and 5 to 150 pixels. Grad-CAM++–defined large-vessel ROIs showed the same directional trend across bands, although the difference was statistically weaker in some alternative band definitions. In contrast, small-vessel VBI differences remained minimal across bands (Supplemental Table 10, available at www.ophthalmologyscience.org).

To assess the effect of image-count imbalance in the R1031C cohort, we performed repeated balanced retraining in which WT training images were repeatedly downsampled to match the number of R1031C training images within each fold while keeping validation and independent test sets fixed. Across 10 repeated retraining runs, image-level soft-ensemble AUC on the hold-out test set was 1.000 at 3-decimal precision in all repeats, and mouse-level soft-ensemble AUC remained 1.000 in all repeats (Supplemental Table 11, available at www.ophthalmologyscience.org).

## Discussion

This study integrates luminal imaging, deep learning, and centerline-based morphometry to characterize CADASIL-related retinal vascular remodeling in vivo. By using FFA, we quantified the perfused lumen, reducing dependence on outer-wall reflectance cues that can confound color fundus photography. Furthermore, continuous centerline sampling enabled detection of spatially structured diameter changes, including diameter CV and VBI that may be diluted by disc-centric sampling.

Human retinal findings in CADASIL appear heterogeneous across modalities and study designs. Conventional fundus examination and fluorescein angiography studies have reported arteriolar narrowing, arteriovenous nicking, peripapillary arteriolar sheathing, cotton-wool spots, and occasional arterial tortuosity, whereas overt angiographic hypoperfusion has generally not been a dominant finding.^3^, ^4^, ^5^, ^6^, ^7^ Multimodal OCT-based analyses have also reported venous dilation and increased outer arterial and venous diameters with increased vessel wall thickness, while OCT angiography studies have emphasized reduced retinal vessel density, especially in deep or macular plexus-based measures.^7^^,^^27^^,^^28^ More recent adaptive-optics imaging has further shown increased wall-to-lumen ratio, smaller arterial internal diameter, and impaired light-induced arterial dilation, indicating structural wall remodeling and impaired vasoreactivity in human CADASIL.^29^ At the same time, ocular histopathology has demonstrated arterial wall thickening, fibrosis, VSMC loss, GOM deposition, and pericyte degeneration in retinal and related ocular vessels.^2^^,^^30^ We therefore interpret our mouse findings not as a direct one-to-one reproduction of all human retinal CADASIL imaging findings but rather as a candidate segmental luminal remodeling phenotype that is biologically plausible in the context of CADASIL vascular wall disease and may be diluted in whole-field or plexus-averaged human analyses. Direct translational validation in human FFA, OCT angiography, and high-resolution structural retinal imaging data sets will be required.

Across both *NOTCH3* knock-in lines, whole-image analysis consistently captured generalized large-vessel dilation and reduced tortuosity. However, patterns differed in the microvasculature. C455R showed a modest increase in whole-image small-vessel diameter CV, whereas R1031C showed no significant whole-image differences in small vessels. These findings suggest that diffuse small-vessel remodeling is subtle in this data set, although global large-vessel dilation and straightening were shared across genotypes.

In contrast, restricting morphometry to XAI-defined ROIs derived from Grad-CAM++ and occlusion amplified focal pathology. In both C455R and R1031C, XAI-restricted large-vessel segments showed increases in maximum diameter and VBI beyond what was detected by whole-image averaging. This suggests that discriminative morphometric signals are spatially concentrated and can be masked by global averaging, even with centerline-based sampling, because focal abnormal segments are embedded within a larger background of relatively preserved vessels. Moreover, convergence of findings across a gradient-based method, Grad-CAM++, and a perturbation-based method, occlusion sensitivity, increases confidence that these focal phenotypes reflect true biological pathology rather than artifacts of a single explanation technique. Given that saliency maps can vary with modeling choices and should not be interpreted as causal evidence,^25^^,^^26^ we interpret XAI as hypothesis-generating and prioritize endpoints that are consistent across methods.

Diameter irregularity showed allele-dependent and method-dependent nuances. In C455R, diameter CV increased consistently within large-vessel ROIs across both XAI methods. In R1031C, diameter CV increased in occlusion-defined ROIs but did not reach significance in Grad-CAM++ ROIs. This pattern suggests that while VBI showed the same directional pattern across methods, dispersion-based descriptors such as CV may be more sensitive to ROI definition. In this setting, VBI may provide a more stable marker of periodic change of vessel diameter in CADASIL.

Large vessels exhibited more pronounced remodeling than small vessels. This compartmental difference is biologically plausible because CADASIL is primarily a mural-cell and VSMC arteriopathy.^1^^,^^30^ Consistent with this interpretation, ocular histopathology in CADASIL has reported arterial wall thickening with fibrosis, VSMC loss, and GOM deposition in retinal arteries, with relative sparing of the choroid.^2^ Vascular smooth muscle cells provide continuous coverage in larger arterioles, whereas pericytes predominate in the capillary bed, offering a plausible substrate for stronger large-vessel beading and dilation signals in our analysis.

Our goal was not to replace explicit morphometric analysis with deep learning but to use explainable deep learning as a localization step that identifies disease-informative vessel regions. Quantification itself remained based on explicit centerline-derived morphometric measurements.

To characterize diameter variation, we employed 2 complementary descriptors, diameter CV and VBI. While diameter CV summarizes dispersion, it lacks spatial context. Vessel beading index specifically targets periodic oscillatory constrictions and dilations. Frequency-domain descriptors have been applied to quantify venous beading in diabetic retinopathy.^11^^,^^12^ We speculate that CADASIL-associated beading reflects a distinct mechanism in which segmental loss of contractile mural function and subsequent fibrosis produce alternating zones of dilation and constriction.^1^^,^^2^ The strong VBI elevation within XAI-defined regions, particularly in large vessels, suggests that periodic patterning may represent a key morphological signature captured by the classifier.

We also observed reduced whole-field large-vessel tortuosity. Prior reports in CADASIL patients describe heterogeneous retinal vascular geometry, with findings varying by methodology.^3^, ^4^, ^5^, ^6^, ^7^ In this mouse FFA pipeline, decreased tortuosity may reflect increased mural stiffness due to chronic remodeling. Mechanistic interpretation remains indirect without spatially matched histology or longitudinal follow-up. Vessel beading index should therefore be viewed as a novel candidate morphometric biomarker complementary to previously reported caliber-based, tortuosity-based, vessel density–based, wall thickness–based, and vasoreactivity-based retinal measures rather than as an already established human CADASIL retinal marker.^3^, ^4^, ^5^, ^6^, ^7^^,^^27^, ^28^, ^29^

The added robustness analyses support that the principal large-vessel signal is not explained by arbitrary analysis choices. In the C455R cohort, large-vessel VBI remained higher across alternative spatial-period bands, with strongest support in Grad-CAM++ ROIs and concordant directional support in occlusion-defined ROIs. In both mutant cohorts, the large-vessel phenotype also remained directionally consistent under alternative ROI thresholds and under out-of-sample saliency estimation. In the smaller R1031C cohort, repeated balanced retraining further indicated that the principal classification signal was not driven solely by the larger WT image pool. Our study has limitations. Sample sizes were small, and age and sex distributions were not fully balanced across genotypes, precluding robust assessment of sex-specific effects. Future studies should include balanced male and female cohorts and prespecify sex-stratified analyses. Formal sex-stratified analysis was not feasible because the WT cohort contained no female mice. However, the shared directional large-vessel phenotype across the 2 independent mutant lines provides indirect reassurance that this phenotype is unlikely to be explained solely by sex or age differences, although confounding cannot be excluded. Additionally, the current pipeline does not distinguish arteries from veins, preventing vessel-type–specific analyses. Cerebral autosomal dominant arteriopathy with subcortical infarcts and leukoencephalopathy is primarily characterized as an arteriopathy, but reliable artery–vein classification in mouse imaging was challenging because very early-phase FFA was not consistently available. Even with cross-referencing to color fundus photographs, a subset of vessels remained ambiguous, as confirmed by cross-review including verification by H.L., N.R., and K.B. Future work with optimized early arterial filling acquisition and validated vessel-type segmentation may enable robust artery–vein–specific quantification. Because all mice were imaged under the same anesthesia protocol, systematic bias across genotypes should be reduced. However, anesthesia may still influence retinal vascular tone and therefore absolute vascularity metrics. Although the repeated balanced retraining analyses indicate that the high WT-versus-R1031C classification performance is not solely attributable to the larger WT image pool, the smaller and demographically imbalanced R1031C cohort remains an important limitation. Finally, VGG16 was chosen as a pragmatic architecture for a moderate-sized data set and for compatibility with Grad-CAM++ visualization; thus, the present conclusions should not be interpreted as architecture-independent, and future benchmarking against newer backbones would be valuable.

## Conclusions

Deep learning models trained on CADASIL FFA images highlight focal vascular dilation and periodic beading. While these pathological features are diluted in whole-image averages, they are robustly detected within AI-identified regions using centerline-based spectral morphometry. This integrated framework offers an interpretable approach for identifying retinal biomarkers of small-vessel disease with potential translational relevance to human CADASIL.
