# Supplementary material for: Deep Learning–Guided Retinal Vascular Morphometric Quantification in Cerebral Autosomal Dominant Arteriopathy with Subcortical Infarcts and Leukoencephalopathy Mouse Models
Source: Ophthalmol Sci. 2026 Jun 10;6(8):101279. doi: 10.1016/j.xops.2026.101279 (PMC13380752; doi:10.1016/j.xops.2026.101279)
Supplement: Supplementary Tables [file mmc1.pdf]

Supplemental Table 1. Summary of animals and FFA images included in the study

Strain	Age (months)	No. of animals	Number of FFA images	Sex (M/F)
WT	6	12	711	12/0
C455R	6	12	810	9/3
R1031C	8	8	149	2/6

WT, wild-type; FFA, fundus fluorescein angiography. C455R and R1031C denote CADASIL knock-in mouse lines carrying the human $NOTCH3^{C455R}$ and $NOTCH3^{R1031C}$ mutants, respectively.

Supplemental Table 2. Independent hold-out test-set classification summary

Comparison	Test mice	Test images	Image soft AUC	Image weighted AUC	Image hard-vote AUC	Mouse soft AUC	Mouse weighted AUC	Mouse hard-vote AUC
WT vs C455R	4 (WT 2, C455R 2)	257	0.984	0.984	0.954	1.000	1.000	1.000
WT vs R1031C	3 (WT 1, R1031C 2)	95	1.000	1.000	1.000	1.000	1.000	1.000

Classification performance was evaluated using only the independent hold-out test mice from the original mouse-level grouped split. Soft, weighted, and hard-vote ensemble predictions are summarized at both image and mouse levels. Mouse-level performance was obtained by aggregating image-level predictions within each mouse. AUC, area under the receiver operating characteristic curve.

Supplemental Table 3. Quantitative analysis of large and small retinal vessels in FFA images (whole-image)

Vessel type	Measure	WT	C455R	R1031C	P (WT vs C455R)	Mean difference (95% CI), WT vs C455R	P (WT vs R1031C)	Mean difference (95% CI), WT vs R1031C
Large vessels	Mean diameter (µm)	27.820 ± 1.283	30.937 ± 2.209	32.033 ± 2.238	0.001*	3.118 (1.797 to 4.573)	<0.001*	4.213 (2.540 to 5.784)
	Maximum diameter (µm)	70.570 ± 3.130	76.223 ± 10.194	74.658 ± 5.743	0.089	5.652 (0.002 to 11.736)	0.135	4.088 (0.066 to 8.254)
	Diameter CV	0.297 ± 0.020	0.294 ± 0.028	0.283 ± 0.022	0.624	-0.003 (-0.021 to 0.016)	0.115	-0.014 (-0.030 to 0.005)
	VBI	2.947 ± 0.410	3.541 ± 0.732	3.121 ± 0.273	0.040	0.594 (0.171 to 1.073)	0.473	0.174 (-0.107 to 0.466)
	Tortuosity	1.076 ± 0.003	1.066 ± 0.003	1.066 ± 0.002	<0.001*	-0.010 (-0.012 to -0.007)	<0.001*	-0.009 (-0.012 to -0.007)
Small vessels	Mean diameter (µm)	14.258 ± 0.392	14.902 ± 0.860	14.369 ± 0.791	0.030	0.645 (0.158 to 1.200)	1.000	0.112 (-0.429 to 0.671)
	Maximum diameter (µm)	40.741 ± 3.136	44.528 ± 4.756	40.455 ± 2.783	0.046	3.787 (0.957 to 7.074)	0.792	-0.286 (-2.750 to 2.245)
	Diameter CV	0.352 ± 0.013	0.367 ± 0.014	0.346 ± 0.006	0.017*	0.015 (0.006 to 0.026)	0.181	-0.006 (-0.014 to 0.002)
	VBI	1.050 ± 0.173	1.133 ± 0.278	1.071 ± 0.194	0.237	0.083 (-0.099 to 0.254)	0.970	0.021 (-0.133 to 0.174)
	Tortuosity	1.140 ± 0.023	1.139 ± 0.022	1.122 ± 0.009	0.795	-0.001 (-0.018 to 0.016)	0.039	-0.018 (-0.032 to -0.005)

Values are subject-level means ± SD. P values were determined by Mann–Whitney U tests comparing WT vs. Mutant. Mean differences are reported as mutant minus WT with bootstrap 95% confidence intervals. An asterisk (*) indicates statistical significance after Bonferroni correction (P < 0.025). CV, coefficient of variation; VBI, Vessel Beading Index. C455R and R1031C denote CADASIL knock-in mouse lines carrying the human $NOTCH3^{C455R}$ and $NOTCH3^{R1031C}$ mutants, respectively. Large-vessel mean diameter, maximum diameter, and VBI were the pre-designated principal morphometric end points of interest; the remaining measures are secondary/supportive.

Supplemental Table 4. XAI-restricted vascular metrics (Grad-CAM++ ROIs)

Vessel type	Measure	WT	C455R	R1031C	P (WT vs C455R)	Mean difference (95% CI), WT vs C455R	P (WT vs R1031 C)	Mean difference (95% CI), WT vs R1031C
Large vessels	Mean diameter (µm)	25.043 ± 1.942	30.703 ± 3.359	31.795 ± 2.746	<0.001*	5.661 (3.512 to 7.802)	<0.001*	6.753 (4.689 to 8.786)
	Maximum diameter (µm)	37.617 ± 2.838	48.463 ± 6.234	45.657 ± 3.823	<0.001*	10.847 (7.069 to 14.512)	<0.001*	8.041 (5.316 to 11.192)
	Diameter CV	0.220 ± 0.037	0.265 ± 0.032	0.250 ± 0.048	0.006*	0.046 (0.020 to 0.073)	0.305	0.030 (-0.006 to 0.069)
	VBI	1.745 ± 0.455	3.041 ± 1.003	2.933 ± 0.562	0.001*	1.296 (0.698 to 1.907)	<0.001*	1.189 (0.738 to 1.616)
	Tortuosity	1.087 ± 0.009	1.070 ± 0.013	1.076 ± 0.014	0.003*	-0.017 (-0.025 to -0.007)	0.098	-0.011 (-0.021 to 0.000)
Small vessels	Mean diameter (µm)	13.814 ± 0.834	14.510 ± 1.168	14.238 ± 0.533	0.078	0.696 (-0.056 to 1.519)	0.098	0.424 (-0.175 to 0.958)
	Maximum diameter (µm)	31.997 ± 3.301	34.690 ± 3.947	33.167 ± 1.706	0.100	2.693 (-0.045 to 5.519)	0.396	1.170 (-0.995 to 3.246)
	Diameter CV	0.337 ± 0.024	0.359 ± 0.025	0.338 ± 0.017	0.046	0.022 (0.003 to 0.040)	0.851	0.000 (-0.017 to 0.017)
	VBI	0.995 ± 0.306	1.208 ± 0.556	1.080 ± 0.331	0.371	0.213 (-0.129 to 0.555)	0.571	0.085 (-0.187 to 0.355)
	Tortuosity	1.134 ± 0.016	1.144 ± 0.030	1.120 ± 0.022	0.795	0.010 (-0.008 to 0.029)	0.115	-0.015 (-0.031 to 0.002)

Values are subject-level means ± SD. P values were determined by Mann–Whitney U tests comparing WT vs. Mutant. Mean differences are reported as mutant minus WT with bootstrap 95% confidence intervals. An asterisk (*) indicates statistical significance after Bonferroni correction (P < 0.025). CV, coefficient of variation; VBI, Vessel Beading Index. C455R and R1031C denote CADASIL knock-in mouse lines carrying the human $NOTCH3^{C455R}$ and $NOTCH3^{R1031C}$ mutants, respectively. Large-vessel mean diameter, maximum diameter, and VBI were the pre-designated principal morphometric end points of interest; the remaining measures are secondary/supportive.

Supplemental Table 5. XAI-restricted vascular metrics (Occlusion ROIs)

Vessel type	Measure	WT	C455R	R1031C	P (WT vs C455R)	Mean difference (95% CI), WT vs C455R	P (WT vs R1031C)	Mean difference (95% CI), WT vs R1031C
Large vessels	Mean diameter (μm)	24.109 ± 1.680	27.953 ± 2.411	30.125 ± 2.246	0.001*	3.844 (2.229 to 5.434)	<0.001*	6.015 (4.266 to 7.690)
	Maximum diameter (μm)	36.501 ± 2.483	47.864 ± 6.492	47.388 ± 3.860	<0.001*	11.363 (7.481 to 15.101)	<0.001*	10.887 (8.006 to 13.604)
	Diameter CV	0.225 ± 0.037	0.278 ± 0.042	0.287 ± 0.020	0.009*	0.053 (0.024 to 0.085)	<0.001*	0.062 (0.038 to 0.085)
	VBI	2.112 ± 0.604	3.835 ± 1.417	4.897 ± 0.869	<0.001*	1.723 (0.925 to 2.598)	<0.001*	2.785 (2.135 to 3.441)
	Tortuosity	1.091 ± 0.007	1.077 ± 0.009	1.088 ± 0.007	0.001*	-0.014 (-0.020 to -0.008)	0.521	-0.003 (-0.009 to 0.002)
Small vessels	Mean diameter (μm)	13.840 ± 0.707	14.272 ± 0.669	13.978 ± 1.064	0.126	0.432 (-0.120 to 0.932)	0.678	0.138 (-0.683 to 0.897)
	Maximum diameter (μm)	33.380 ± 2.772	34.936 ± 3.225	32.227 ± 4.356	0.149	1.556 (-0.767 to 3.848)	0.270	-1.153 (-4.245 to 2.179)
	Diameter CV	0.342 ± 0.020	0.355 ± 0.018	0.338 ± 0.032	0.089	0.014 (-0.001 to 0.028)	0.970	-0.003 (-0.027 to 0.019)
	VBI	0.981 ± 0.242	1.072 ± 0.365	0.899 ± 0.319	0.583	0.091 (-0.147 to 0.324)	0.473	-0.081 (-0.326 to 0.162)
	Tortuosity	1.141 ± 0.016	1.145 ± 0.030	1.154 ± 0.049	0.751	0.003 (-0.014 to 0.022)	0.910	0.012 (-0.018 to 0.047)

Analysis was restricted to regions identified by occlusion sensitivity maps. Values are subject-level means ± SD. P values were determined by Mann–Whitney U tests comparing WT vs. Mutant. Mean differences are reported as mutant minus WT with bootstrap 95% confidence intervals. An asterisk (*) indicates statistical significance after Bonferroni correction (P < 0.025). CV, coefficient of variation; VBI, Vessel Beading Index; ROI, region of interest. C455R and R1031C denote CADASIL knock-in mouse lines carrying the human $NOTCH3^{C455R}$ and $NOTCH3^{R1031C}$ mutants, respectively. Large-vessel mean diameter, maximum diameter, and VBI were the pre-designated principal morphometric end points of interest; the remaining measures are secondary/supportive.

Supplemental Table 6. Out-of-sample ROI-restricted vascular metrics

Panel A. WT vs C455R

Method	Vessel type	Measure	WT mean ± SD	C455R mean ± SD	Mean difference (95% CI)	P
GradCAM++	Large	Mean diameter (μm)	25.083 ± 1.818	30.683 ± 3.487	5.600 (3.507 to 7.775)	<0.001
		Maximum diameter (μm)	37.821 ± 4.555	45.120 ± 6.279	7.299 (3.145 to 11.436)	0.005
		Diameter CV	0.219 ± 0.053	0.248 ± 0.038	0.028 (-0.006 to 0.065)	0.126
		Tortuosity	1.085 ± 0.010	1.083 ± 0.017	-0.002 (-0.012 to 0.009)	0.312
		VBI	1.843 ± 0.755	3.520 ± 0.752	1.677 (1.100 to 2.245)	<0.001
GradCAM++	Small	Mean diameter (μm)	13.500 ± 0.770	14.358 ± 0.779	0.858 (0.237 to 1.434)	0.010
		Maximum diameter (μm)	30.487 ± 3.644	32.189 ± 2.212	1.702 (-0.719 to 3.986)	0.157
		Diameter CV	0.329 ± 0.023	0.348 ± 0.021	0.019 (0.002 to 0.036)	0.053
		Tortuosity	1.143 ± 0.028	1.152 ± 0.037	0.009 (-0.015 to 0.035)	0.403
		VBI	0.988 ± 0.317	1.185 ± 0.490	0.197 (-0.110 to 0.521)	0.285
Occlusion	Large	Mean diameter (μm)	24.017 ± 1.984	27.420 ± 2.461	3.403 (1.773 to 5.097)	0.002
		Maximum diameter (μm)	36.426 ± 3.982	42.736 ± 5.973	6.310 (2.541 to 10.241)	0.012
		Diameter CV	0.228 ± 0.048	0.258 ± 0.043	0.031 (-0.003 to 0.067)	0.175
		Tortuosity	1.096 ± 0.014	1.087 ± 0.013	-0.009 (-0.020 to 0.001)	0.175
		VBI	2.561 ± 1.331	3.724 ± 0.897	1.163 (0.237 to 1.947)	0.026
Occlusion	Small	Mean diameter (μm)	13.565 ± 0.621	14.541 ± 0.978	0.976 (0.365 to 1.650)	0.007
		Maximum diameter (μm)	31.069 ± 3.842	33.194 ± 3.786	2.125 (-0.971 to 5.060)	0.100
		Diameter CV	0.332 ± 0.021	0.353 ± 0.019	0.020 (0.005 to 0.036)	0.040
		Tortuosity	1.156 ± 0.020	1.171 ± 0.069	0.015 (-0.019 to 0.058)	0.931
		VBI	1.066 ± 0.302	1.047 ± 0.278	-0.019 (-0.243 to 0.202)	0.795

Panel B. WT vs R1031C

Method	Vessel type	Measure	WT mean ± SD	R1031C mean ± SD	Mean difference (95% CI)	P
GradCAM++	Large	Mean diameter (µm)	25.525 ± 2.675	31.886 ± 3.198	6.361 (3.703 to 8.821)	0.001
		Maximum diameter (µm)	37.035 ± 2.857	44.765 ± 3.391	7.730 (4.987 to 10.495)	<0.001
		Diameter CV	0.209 ± 0.037	0.214 ± 0.022	0.005 (-0.020 to 0.030)	0.571
		Tortuosity	1.087 ± 0.007	1.073 ± 0.009	-0.014 (-0.021 to -0.007)	0.002
		VBI	2.363 ± 0.912	3.374 ± 0.943	1.011 (0.252 to 1.781)	0.025
GradCAM++	Small	Mean diameter (µm)	13.940 ± 0.933	14.398 ± 0.527	0.458 (-0.183 to 1.031)	0.181
		Maximum diameter (µm)	31.671 ± 4.673	31.484 ± 1.866	-0.187 (-3.100 to 2.575)	1.000
		Diameter CV	0.348 ± 0.036	0.331 ± 0.022	-0.017 (-0.041 to 0.008)	0.521
		Tortuosity	1.135 ± 0.014	1.114 ± 0.019	-0.021 (-0.036 to -0.007)	0.012
		VBI	1.064 ± 0.244	1.080 ± 0.610	0.017 (-0.329 to 0.495)	0.343
Occlusion	Large	Mean diameter (µm)	24.481 ± 2.123	28.290 ± 2.889	3.808 (1.687 to 6.078)	0.007
		Maximum diameter (µm)	37.793 ± 4.085	44.456 ± 5.363	6.663 (2.731 to 10.782)	0.003
		Diameter CV	0.223 ± 0.040	0.276 ± 0.031	0.053 (0.023 to 0.082)	0.007
		Tortuosity	1.089 ± 0.012	1.093 ± 0.010	0.004 (-0.005 to 0.013)	0.521
		VBI	2.504 ± 0.960	4.673 ± 0.966	2.169 (1.370 to 2.970)	<0.001
Occlusion	Small	Mean diameter (µm)	13.806 ± 0.877	13.495 ± 0.930	-0.312 (-1.070 to 0.459)	0.473
		Maximum diameter (µm)	31.437 ± 3.786	29.253 ± 3.576	-2.184 (-5.292 to 0.823)	0.231
		Diameter CV	0.347 ± 0.025	0.330 ± 0.038	-0.017 (-0.046 to 0.012)	0.238
		Tortuosity	1.153 ± 0.030	1.143 ± 0.036	-0.010 (-0.037 to 0.020)	0.238
		VBI	0.961 ± 0.316	0.858 ± 0.433	-0.103 (-0.432 to 0.222)	0.521

ROI-restricted morphometry was recalculated under the out-of-sample saliency policy, such that each

saliency map was generated only with models that had not been trained on the corresponding mouse. Values are subject-level means ± SD. Mean differences are reported as mutant minus WT with bootstrap 95% confidence intervals. Exact two-sided Mann–Whitney U P values are shown because these analyses are presented as supportive sensitivity analyses. CV, coefficient of variation; VBI, Vessel Beading Index; ROI, region of interest. P values are reported to three decimal places, with values <0.001 shown as <0.001. Large-vessel mean diameter, maximum diameter, and VBI were the predefined principal morphometric end points of interest; the remaining measures are secondary/supportive.

Supplemental Table 7. C455R threshold sensitivity of large-vessel ROI-restricted morphometry

Panel A. Grad-CAM++ large-vessel ROIs

Metric	Threshold percentile	WT mean ± SD	C455R mean ± SD	Mean difference (95% CI)	P
Mean diameter (μm)	80	25.625 ± 1.947	30.351 ± 2.318	4.726 (3.111 to 6.327)	<0.001
	85	25.234 ± 1.729	30.622 ± 2.605	5.389 (3.754 to 7.049)	<0.001
	90	25.083 ± 1.818	30.683 ± 3.487	5.600 (3.507 to 7.775)	<0.001
	95	25.204 ± 2.492	30.000 ± 4.978	4.796 (1.722 to 7.815)	0.007
	97.5	23.928 ± 3.378	28.873 ± 5.025	4.945 (1.628 to 8.343)	0.015
Maximum diameter (μm)	80	39.849 ± 4.161	47.544 ± 6.084	7.695 (3.699 to 11.595)	0.004
	85	38.981 ± 4.678	47.364 ± 6.166	8.383 (4.209 to 12.571)	0.002
	90	37.821 ± 4.555	45.120 ± 6.279	7.299 (3.145 to 11.436)	0.005
	95	35.675 ± 4.007	42.097 ± 7.504	6.422 (1.835 to 11.126)	0.017
	97.5	32.876 ± 5.430	39.445 ± 7.415	6.569 (1.474 to 11.587)	0.021
VBI	80	1.885 ± 0.455	3.321 ± 0.982	1.436 (0.857 to 2.033)	<0.001
	85	1.892 ± 0.493	3.525 ± 0.922	1.634 (1.056 to 2.206)	<0.001
	90	1.843 ± 0.755	3.520 ± 0.752	1.677 (1.100 to 2.245)	<0.001
	95	2.483 ± 1.413	3.866 ± 1.108	1.383 (0.362 to 2.297)	0.014
	97.5	2.479 ± 1.581	5.276 ± 3.558	2.798 (0.745 to 5.008)	0.025

Panel B. Occlusion-defined large-vessel ROIs

Metric	Threshold percentile	WT mean ± SD	C455R mean ± SD	Mean difference (95% CI)	P
Mean diameter (μm)	80	24.401 ± 1.339	27.154 ± 2.006	2.753 (1.450 to 4.064)	0.002
	85	24.019 ± 1.544	27.284 ± 2.061	3.265 (1.909 to 4.736)	<0.001
	90	24.017 ± 1.984	27.420 ± 2.461	3.403 (1.773 to 5.097)	0.002
	95	23.478 ± 2.769	26.851 ± 4.342	3.373 (0.508 to 6.158)	0.023
	97.5	22.936 ± 2.971	26.254 ± 4.046	3.318 (0.617 to 5.935)	0.040
Maximum diameter (μm)	80	38.361 ± 3.498	46.028 ± 6.564	7.668 (3.783 to 11.612)	0.003
	85	37.700 ± 3.268	45.312 ± 7.169	7.613 (3.551 to 12.038)	0.010

Metric	Threshold percentile	WT mean ± SD	C455R mean ± SD	Mean difference (95% CI)	P
	90	36.426 ± 3.982	42.736 ± 5.973	6.310 (2.541 to 10.241)	0.012
	95	33.236 ± 4.540	40.476 ± 8.848	7.240 (1.883 to 12.509)	0.009
	97.5	30.117 ± 4.067	36.996 ± 8.942	6.879 (1.430 to 12.050)	0.061
VBI	80	2.250 ± 0.777	4.158 ± 0.550	1.908 (1.385 to 2.388)	<0.001
	85	2.367 ± 0.892	3.766 ± 0.847	1.400 (0.703 to 2.049)	0.002
	90	2.561 ± 1.331	3.724 ± 0.897	1.163 (0.237 to 1.947)	0.026
	95	2.480 ± 0.926	3.416 ± 1.596	0.936 (-0.057 to 1.942)	0.214
	97.5	1.981 ± 1.026	3.363 ± 2.266	1.382 (0.147 to 2.796)	0.100

Analysis was restricted to large-vessel ROIs on the final out-of-sample manifest while varying only the saliency threshold (80th, 85th, 90th, 95th, and 97.5th percentiles). VBI was computed using the primary 10–100 pixel spatial-period band. Values are subject-level means ± SD. Mean differences are reported as mutant minus WT with bootstrap 95% confidence intervals. Exact two-sided Mann–Whitney U P values are shown because these analyses are presented as supportive sensitivity analyses. VBI, vessel beading index. P values are reported to three decimal places, with values <0.001 shown as <0.001.

Supplemental Table 8. C455R VBI sensitivity across alternative spatial-period bands

Method	Vessel type	Band (pixels)	WT mean ± SD	C455R mean ± SD	Mean difference (95% CI)	P
GradCAM++	Large	10-100	1.843 ± 0.755	3.520 ± 0.752	1.677 (1.100 to 2.245)	<0.001
		15-100	1.660 ± 0.710	3.258 ± 0.764	1.598 (1.026 to 2.162)	<0.001
		20-80	1.348 ± 0.701	2.554 ± 0.711	1.206 (0.651 to 1.741)	0.001
		5-150	2.247 ± 0.835	4.152 ± 0.867	1.906 (1.200 to 2.502)	<0.001
GradCAM++	Small	10-100	0.988 ± 0.317	1.185 ± 0.490	0.197 (-0.110 to 0.521)	0.285
		15-100	0.714 ± 0.284	0.884 ± 0.461	0.170 (-0.116 to 0.473)	0.312
		20-80	0.458 ± 0.224	0.561 ± 0.336	0.103 (-0.104 to 0.335)	0.371
		5-150	1.235 ± 0.324	1.462 ± 0.513	0.228 (-0.087 to 0.565)	0.341
Occlusion	Large	10-100	2.561 ± 1.331	3.724 ± 0.897	1.163 (0.237 to 1.947)	0.026
		15-100	2.370 ± 1.303	3.376 ± 0.883	1.006 (0.084 to 1.794)	0.030
		20-80	1.965 ± 1.200	2.900 ± 0.715	0.934 (0.100 to 1.612)	0.014
		5-150	3.007 ± 1.370	4.564 ± 1.118	1.557 (0.538 to 2.450)	0.019
Occlusion	Small	10-100	1.066 ± 0.302	1.047 ± 0.278	-0.019 (-0.243 to 0.202)	0.795
		15-100	0.795 ± 0.306	0.769 ± 0.273	-0.027 (-0.253 to 0.190)	0.931
		20-80	0.504 ± 0.218	0.554 ± 0.402	0.050 (-0.187 to 0.313)	0.751
		5-150	1.331 ± 0.321	1.327 ± 0.291	-0.004 (-0.241 to 0.228)	0.795

VBI sensitivity analysis was performed on the final out-of-sample manifest with the XAI ROI threshold fixed at the primary 90th-percentile setting. Values are subject-level means ± SD. Mean differences are reported as mutant minus WT with bootstrap 95% confidence intervals. Exact two-sided Mann–Whitney U P values are shown because these analyses are presented as supportive sensitivity analyses. Both large- and small-vessel rows are included to document the large-vessel-dominant nature of the band-sensitive signal. VBI, Vessel Beading Index. P values are reported to three decimal places, with values <0.001 shown as <0.001.

Supplemental Table 9. R1031C threshold sensitivity of large-vessel ROI-restricted morphometry

Panel A. Grad-CAM++ large-vessel ROIs

Metric	Threshold percentile	WT mean ± SD	R1031C mean ± SD	Mean difference (95% CI)	P
Mean diameter (μm)	80	25.699 ± 1.984	30.954 ± 2.707	5.255 (3.158 to 7.200)	<0.001
	85	25.707 ± 2.124	31.325 ± 2.412	5.617 (3.664 to 7.473)	<0.001
	90	25.525 ± 2.675	31.886 ± 3.198	6.361 (3.703 to 8.821)	0.001
	95	24.639 ± 3.447	31.162 ± 3.497	6.524 (3.386 to 9.356)	0.002
	97.5	24.245 ± 3.545	28.801 ± 3.513	4.556 (1.503 to 7.510)	0.009
Maximum diameter (μm)	80	39.823 ± 2.710	48.133 ± 3.350	8.310 (5.636 to 10.856)	<0.001
	85	38.942 ± 2.875	46.540 ± 3.230	7.597 (4.924 to 10.232)	<0.001
	90	37.035 ± 2.857	44.765 ± 3.391	7.730 (4.987 to 10.495)	<0.001
	95	33.601 ± 4.674	42.286 ± 4.389	8.685 (4.861 to 12.463)	<0.001
	97.5	30.759 ± 4.714	38.522 ± 4.321	7.763 (3.832 to 11.426)	0.003
VBI	80	1.868 ± 0.724	3.027 ± 0.784	1.159 (0.505 to 1.775)	0.005
	85	2.208 ± 0.843	3.280 ± 0.846	1.072 (0.351 to 1.755)	0.020
	90	2.363 ± 0.912	3.374 ± 0.943	1.011 (0.252 to 1.781)	0.025
	95	2.781 ± 1.196	4.997 ± 1.810	2.216 (0.847 to 3.489)	0.007
	97.5	3.359 ± 2.731	6.404 ± 2.599	3.045 (0.683 to 5.253)	0.020

Panel B. Occlusion-defined large-vessel ROIs

Metric	Threshold percentile	WT mean ± SD	R1031C mean ± SD	Mean difference (95% CI)	P
Mean diameter (μm)	80	24.632 ± 1.775	28.582 ± 2.370	3.950 (2.242 to 5.814)	<0.001
	85	24.728 ± 1.853	28.504 ± 2.221	3.777 (2.086 to 5.578)	0.001
	90	24.481 ± 2.123	28.290 ± 2.889	3.808 (1.687 to 6.078)	0.007
	95	24.992 ± 2.306	28.657 ± 2.643	3.665 (1.607 to 5.787)	0.005
	97.5	24.311 ± 2.762	29.015 ± 3.919	4.704 (1.866 to 7.657)	0.004
Maximum diameter (μm)	80	40.092 ± 4.919	45.874 ± 5.053	5.782 (1.540 to 10.005)	0.012

Metric	Threshold percentile	WT mean ± SD	R1031C mean ± SD	Mean difference (95% CI)	P
	85	39.203 ± 4.694	45.437 ± 5.516	6.234 (1.954 to 10.523)	0.010
	90	37.793 ± 4.085	44.456 ± 5.363	6.663 (2.731 to 10.782)	0.003
	95	36.369 ± 3.634	43.099 ± 5.830	6.730 (2.579 to 11.197)	0.004
	97.5	32.934 ± 3.715	38.288 ± 4.283	5.354 (2.100 to 8.955)	0.011
VBI	80	2.817 ± 1.319	4.710 ± 1.183	1.892 (0.821 to 2.925)	0.012
	85	2.642 ± 1.172	4.940 ± 0.946	2.298 (1.402 to 3.143)	0.002
	90	2.504 ± 0.960	4.673 ± 0.966	2.169 (1.370 to 2.970)	<0.001
	95	3.164 ± 1.842	5.990 ± 2.293	2.826 (1.063 to 4.638)	0.010
	97.5	3.026 ± 1.766	6.911 ± 2.884	3.885 (1.966 to 6.133)	0.002

Analysis was restricted to large-vessel ROIs on the final out-of-sample manifest while varying only the saliency threshold (80th, 85th, 90th, 95th, and 97.5th percentiles). VBI was computed using the primary 10–100 pixel spatial-period band. Values are subject-level means ± SD. Mean differences are reported as mutant minus WT with bootstrap 95% confidence intervals. Exact two-sided Mann–Whitney U P values are shown because these analyses are presented as supportive sensitivity analyses. VBI, vessel beading index. P values are reported to three decimal places, with values <0.001 shown as <0.001.

Supplemental Table 10. R1031C VBI sensitivity across alternative spatial-period bands

Method	Vessel type	Band (pixels)	WT mean ± SD	R1031C mean ± SD	Mean difference (95% CI)	P
GradCAM++	Large	10-100	2.363 ± 0.912	3.374 ± 0.943	1.011 (0.252 to 1.781)	0.025
		15-100	2.198 ± 0.913	3.189 ± 0.928	0.992 (0.238 to 1.754)	0.031
		20-80	1.772 ± 0.825	2.426 ± 0.962	0.654 (-0.087 to 1.405)	0.181
		5-150	2.820 ± 0.999	3.878 ± 1.199	1.058 (0.149 to 2.025)	0.057
GradCAM++	Small	10-100	1.064 ± 0.244	1.080 ± 0.610	0.017 (-0.329 to 0.495)	0.343
		15-100	0.764 ± 0.263	0.643 ± 0.181	-0.121 (-0.311 to 0.062)	0.270
		20-80	0.508 ± 0.208	0.470 ± 0.159	-0.038 (-0.193 to 0.112)	0.970
		5-150	1.329 ± 0.266	1.328 ± 0.594	-0.001 (-0.343 to 0.468)	0.343
Occlusion	Large	10-100	2.504 ± 0.960	4.673 ± 0.966	2.169 (1.370 to 2.970)	<0.001
		15-100	2.279 ± 0.890	4.387 ± 0.965	2.108 (1.302 to 2.886)	<0.001
		20-80	1.908 ± 0.856	3.714 ± 0.875	1.806 (1.061 to 2.516)	<0.001
		5-150	2.843 ± 0.957	5.175 ± 1.176	2.332 (1.403 to 3.272)	<0.001
Occlusion	Small	10-100	0.961 ± 0.316	0.858 ± 0.433	-0.103 (-0.432 to 0.222)	0.521
		15-100	0.673 ± 0.322	0.649 ± 0.387	-0.024 (-0.332 to 0.280)	0.970
		20-80	0.419 ± 0.194	0.445 ± 0.272	0.026 (-0.179 to 0.234)	1.000
		5-150	1.244 ± 0.329	1.125 ± 0.454	-0.119 (-0.463 to 0.221)	0.384

VBI sensitivity analysis was performed on the final out-of-sample manifest with the XAI ROI threshold fixed at the primary 90th-percentile setting. Values are subject-level means ± SD. Mean differences are reported as mutant minus WT with bootstrap 95% confidence intervals. Exact two-sided Mann–Whitney U P values are shown because these analyses are presented as supportive sensitivity analyses. Both large- and small-vessel rows are included to document the large-vessel-dominant nature of the band-sensitive signal. VBI, Vessel Beading Index. P values are reported to three decimal places, with values <0.001 shown as <0.001.

Supplemental Table 11. Repeated balanced retraining results for the WT-versus-R1031C classification task

Repeat	Image soft AUC	Image soft accuracy	Mouse soft AUC	Mouse soft accuracy	Mean validation image accuracy across folds	SD of validation image accuracy across folds	One-class validation folds (n)
1	1.000	0.884	1.000	1.000	0.988	0.020	2
2	1.000	0.937	1.000	1.000	1.000	0.000	2
3	1.000	0.979	1.000	1.000	0.970	0.032	2
4	1.000	0.916	1.000	1.000	0.986	0.020	2
5	1.000	0.958	1.000	1.000	0.971	0.050	2
6	1.000	0.905	1.000	1.000	0.993	0.013	2
7	1.000	0.979	1.000	1.000	0.985	0.025	2
8	1.000	0.926	1.000	1.000	0.994	0.009	2
9	1.000	0.958	1.000	1.000	0.991	0.021	2
10	1.000	0.937	1.000	1.000	0.996	0.004	2

For each repeat and fold, WT training images were downsampled only within the training pool while the original mouse-level grouped split and fixed independent hold-out test set were preserved. Downsampling used a subject-aware random resampling strategy with repeat- and fold-specific fixed seeds. Test-set metrics were computed on the fixed hold-out test set. Some validation folds contained only one class because of the grouped split in a small cohort; in those cases, validation AUC was not defined and is therefore not reported as a primary summary metric.
